# Does a “Western Lifestyle” Confer a Higher Burden of Colorectal Cancer? A Comparison of EU15+ Countries versus Global Trends between 1990 and 2019

**DOI:** 10.3390/cancers16122277

**Published:** 2024-06-19

**Authors:** Bradley Walker, Chinmay T. Jani, Weitao Liu, Shoheera Punjwani, Samuel Kareff, Peter Ceglowski, Harpreet Singh, Melissa Mariano, Justin D. Salciccioli, Lawrence Borges, Gilberto Lopes

**Affiliations:** 1 Department of Medicine, Mount Auburn Hospital, Cambridge, MA 02138, USA; bradley.walker@mah.harvard.edu (B.W.); weitaoliu0605@gmail.com (W.L.); shoheerakhaliqdina@gmail.com (S.P.); peter.ceglowski@mah.harvard.edu (P.C.); melissa.mariano@mah.harvard.edu (M.M.); lawrence.borges@mah.harvard.edu (L.B.); 2 Department of Medicine, Harvard Medical School, Boston, MA 02115, USA; 3 Sylvester Comprehensive Cancer Center, University of Miami, Miami, FL 33136, USA; sak57@med.miami.edu (S.K.); glopes@med.miami.edu (G.L.); 4 Department of Pulmonary and Critical Care, Medical College of Wisconsin, Milwaukee, WI 53226, USA; drhpsingh101@gmail.com; 5 Division of Pulmonary and Critical Care, Brigham and Women’s Hospital, Harvard Medical School, Boston, MA 02115, USA; justin.salciccioli@gmail.com; 6 Division of Gastroenterology, Mount Auburn Hospital, Cambridge, MA 02138, USA

**Keywords:** colorectal cancer, mortality, incidence, GBD, EU 15+, colorectal cancer screening

## Abstract

**Simple Summary:**

In the United States, colorectal cancer (CRC) rates are dropping among adults aged 50 and above, but recent studies show a rise in cases among those under 50. This study compares CRC rates in EU15+ countries to see if similar trends exist in regions with comparable “Western lifestyle” risk factors. Data from 1990 to 2019 were analyzed, focusing on incidence, mortality, and mortality-to-incidence ratios (MIRs) across age groups. Globally, the change in percentage rates of CRC increased for all ages over this 30-year period, especially among males (75.9%) and females (27.7%) aged 25–49. This rise was mirrored in 15 of 19 EU15+ countries for males and 16 for females in the same age group. While female mortality rates decreased globally, male rates increased across all ages, highlighting the need to address modifiable risk factors and implement early standardized screening to detect CRC in younger populations.

**Abstract:**

The incidence of colorectal cancer (CRC) in the U.S. is declining in adults 50 years and older; however, recent studies suggest an increasing disease burden among adults under age 50. This study aims to compare the incidence, mortality, and mortality-to-incidence ratios (MIRs) of CRC in EU15+ countries to determine if similar age-stratified occurrences are observed across these countries with similar “Western lifestyle”-related risk factors. Incidence and mortality rates for CRC between 1990 and 2019 were extracted using the Global Burden of Disease database. The data were age-stratified into groups between ages 25–49, 50–69, and greater than 69 years. We observed that the incidence of CRC increased globally for all age groups, with the highest increase observed for males (75.9%) and females (27.7%) aged 25–49. A similar trend was observed in 15 of the 19 EU15+ countries for males and 16 of the 19 EU15+ countries for females aged 25–49. Global mortality rates decreased for all age groups in females but increased for males in all age groups. This raises concerns regarding potentially modifiable risk factors contributing to increased CRC development and underscores the importance of implementing standardized screening at an earlier stage to ensure adequate detection in the younger population.

## 1. Introduction

Colorectal cancer (CRC) is the third-most diagnosed and the second-most common cause of cancer deaths worldwide [1]. Previous studies have suggested that CRC is the third-most common cancer among men and women globally [2]. In the U.S., CRC remains the third leading cause of cancer-related death among men and women, following lung and prostate cancers in men and lung and breast cancers in women [3]. Recently, there has been a decline in incidence rates of CRC in the U.S., primarily attributed to increased screening rates among adults 50 years or older [1]. Similarly, an EU study found decreasing incidence rates across most countries, with the largest decrease in incidence and mortality in countries with long-standing CRC screening practices [4]. Studies also suggest an increased incidence of CRC among younger U.S. adults under 50 years, which ultimately prompted the United States Preventive Services Task Force (USPSTF) to modify its 2016 guidelines to initiate screening at the age of 45 in 2021 [5]. A study that evaluated the most recent American Cancer Society CRC statistics estimates that 153,020 Americans will be diagnosed with CRC and 52,550 will die from the disease in 2023. Of these patients, 19,550 cases and 3750 deaths will be in patients under 50 years old. This statistical analysis concluded that the CRC burden in the U.S. is shifting to younger patients, with one in five new cases now occurring in individuals in their early fifth decade or younger [1]. A recent retrospective study evaluated new CRC diagnoses, reporting increasing incidence among younger cohorts [6]. Similarly, other studies demonstrated an increasing, age-standardized incidence and mortality of CRC globally [7].

There is a consensus that the promotion of CRC development is largely attributed to various modifiable risk factors in a milieu of underlying and unmodifiable risk factors. While many of these modifiable risk factors may promote CRC development, there is an increased focus on risk factors associated with the “Western lifestyle”, such as obesity, a sedentary lifestyle, and alcohol usage that may be driving this increased incidence, particularly among younger individuals under age 50 [8]. A prior study utilized the Global Burden of Disease (GBD) database to evaluate the age-standardized CRC burden of each country. However, that study did not evaluate age groups of CRC incidence or mortality within higher-income countries, though it did evaluate global incidence based on age groups and concluded that higher increases in incidence were observed in the youngest age bracket below 50 years [9]. The increasing emergence of CRC in this demographic is concerning for establishing these risk factors to optimize mitigation.

This study aims to compare trends in CRC among countries of the European Union (EU) 15+ cohort and global trends during the period ranging from 1990 to 2019 to determine if similar age-stratified occurrences are observed across these countries with predominant “Western lifestyle” influences. Additionally, this study aims to evaluate different age demographics to determine if similar trends are observed, with specific interest in the younger demographic under age 50. The EU15+ cohort includes countries with relatively high incomes and similar healthcare system capacities. This cohort consists of 15 EU countries plus four additional countries, including Australia, Canada, the United Kingdom (UK), and the United States (U.S.). The EU countries include Austria, Belgium, Denmark, Finland, France, Germany, Greece, Ireland, Italy, Luxembourg, Netherlands, Norway, Portugal, Spain, and Sweden. Prior studies have also utilized the EU15+ cohort due to its practicality of geographic comparison as well as the likelihood that these countries exhibit predominantly “Western” lifestyles [10,11]. Data were extracted from the Global Burden of Disease Study (GBD) to evaluate trends in CRC incidence, mortality, and mortality-to-incidence ratios (MIRs) in EU15+ countries and globally from 1990 to 2019.

## 2. Materials and Methods

### 2.1. Characteristics of the Data Source

This study was an observational cross-sectional analysis of age-stratified CRC incidence, mortality, and MIRs among EU15+ countries and global trends using the data from the GBD database. Prior analyses describe the specifics of the methodology utilized by GBD [12,13]. The dataset employed by the GBD collaborators is compiled from a multitude of sources, such as insurance, admission data, outpatient encounters, systematic reviews, registration, autopsy reports, disease registries, surveys, scientific studies, surveillance data, and a host of other sources large and small entered into the GBD. Data sources can be accessed at their website https://ghdx.healthdata.org/gbd-2019 (accessed on 4 May 2024) [14,15]. The GBD database maps all incidence and mortality data related to CRC International Classification of Disease (ICD) codes, including C18-C21, D01.0-D01.3, D37.3-D37.5 ICD-10 codes and 153-154, 230.3-230.6 ICD-9 codes. These data are then combined via Bayesian meta-regression with the DisMord-MR 2.19 tool, which adjusts for bias and yields disease estimates with uncertainty intervals. The quality of the mortality data from each country is evaluated with the GBD methodology in a 5-start system by location and year to assist in the user’s understanding of the reliability of the mortality data. This methodology has been previously used to analyze the EU15+ countries, with 10 of 19 countries scoring five stars, indicating 85–100% completeness of data, and the remaining 9 countries scoring four stars, indicating 65–84% completeness of mortality data [16,17,18].

### 2.2. Handling of the GBD Data

We extracted age-stratified incidence rates (ASIRs) and age-stratified mortality rates (ASMRs) for CRC from EU15+ countries between 1990 and 2019 using the dedicated GBD Study results tool (https://vizhub.healthdata.org/gbd-results/ (accessed on 4 May 2024)). Patients were stratified into age groups of 25–49, 50–69, and greater than 70 years. All rates were reported per 100,000 population. Additionally, the ASIRs and ASMRs for males and females at global level were extracted. By applying the WHO standard population to each country, age-standardized rates are utilized to help adjust for varying age demographics between countries that may introduce bias due to the inherent variability between different countries or geographic regions. Global trends include 204 countries and territories as well as first administrative level disaggregations for 22 countries from 1990 to 2019 [19].

The absolute changes in ASIRs and ASMRs between 1990 and 2019 for each gender in each EU15+ country and globally were calculated. MIRs were calculated by dividing ASMR by ASIR for each year (1990 and 2019) in each EU15+ country and globally. MIRs help to compare disease burden by normalizing mortality to incidence. MIRs are generally used as a comparative indicator of inequities regarding cancer outcomes. The WHO employs this metric to evaluate specific disease burden on a healthcare system [20].

### 2.3. Statistical Analysis

Joinpoint Command Line Version 4.5.0.1 was used to assess the trends of the ASIRs and ASMRs for males and females at global level. This software applies a Joinpoint regression analysis to the datasets to observe data trends over a specific period by connecting trends with the simplest model possible on a logarithmic scale. This methodology allows for establishing inflection points, thus highlighting any change in trends. The simplest model in this approach is represented by a straight line without Joinpoints. As more Joinpoints are added to a dataset, each is tested for significance via a Monte Carlo permutation method. Additionally, the software computes an estimated annual percentage change (EACP) (with 95% confidence intervals) for each Joinpoint line segment with testing for significance. The outcome is a series of statistically significant Joinpoints for males and females at global level with either a positive or negative trend represented by a potentially significant EAPC. This approach allows for the assessment of temporal trends and the comparison of global trends.

## 3. Results:

### 3.1. Trends in CRC ASIRs, 1990–2019

**Figure 1:**
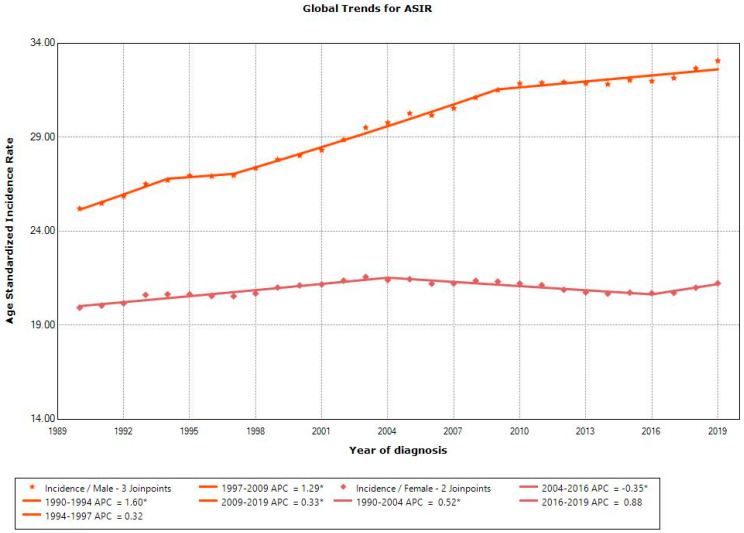
Joinpoint analysis of age-standardized incidence rates for males and females at global level between 1990 and 2019. * indicates a significant *p*-value of <0.05.

The ASIR of CRC increased globally for all age groups over the study period, with the highest increase observed for males (+75.9%) and females (+27.7%) aged 25–49 (Supplementary Table S1). The Joinpoint analysis ASIRs of males at the global level showed a significant increase from 1990 to 2019, while females at the global level showed no significant change (Figure 1).

The youngest age group observed an increasing trend in ASIR, with 17 of the 19 EU15+ countries for males and 16 of the 19 countries for females exhibiting increased ASIRs. Luxembourg and Austria were common countries in both genders of the young age bracket that experienced ASIR reduction. At the same time, Belgium reported decreased ASIR in young females (−2.78%) but an increase in young males (+12.19%).

ASIR increased globally and in most EU15+ countries, which was also observed in the Joinpoint analysis of global trends. However, a few countries exhibited reducing ASIR (likely attributed to the early practice of CRC screening and a high percentage of population screening). ASIR increased globally and in a majority of the EU15+ countries. However, ASIR rates decreased in 8 countries in males aged 50–69 and in 10 countries in females aged 50–69. There were seven common countries between both genders of this age group, including Australia (−15.93% males, −22.57% females), Austria (−40.81% males, 45.64% females), Germany (−2.26% males, −20.06% females), Ireland (−5.13 males, −9.38 females), Luxembourg (−24.85 males, −28.43 females), the United Kingdom (−9.39% males, −15.59% females), and the United States (−11.27% males, −16.97% females). France exhibited a decline in males aged 50–69 (−1.95%) but an increase in ASIR in females aged 50–69 (+4.02%). Three countries reported decreasing ASIR in females of this age group but an increase in their male counterparts: Belgium (+0.58% males, −13.54% females), Canada (+0.83% males, −4.59% females), and Sweden (+2.73% males, −5.39% females). All other countries for both males and females aged 50–69 exhibited increased ASIRs.

**Figure 2:**
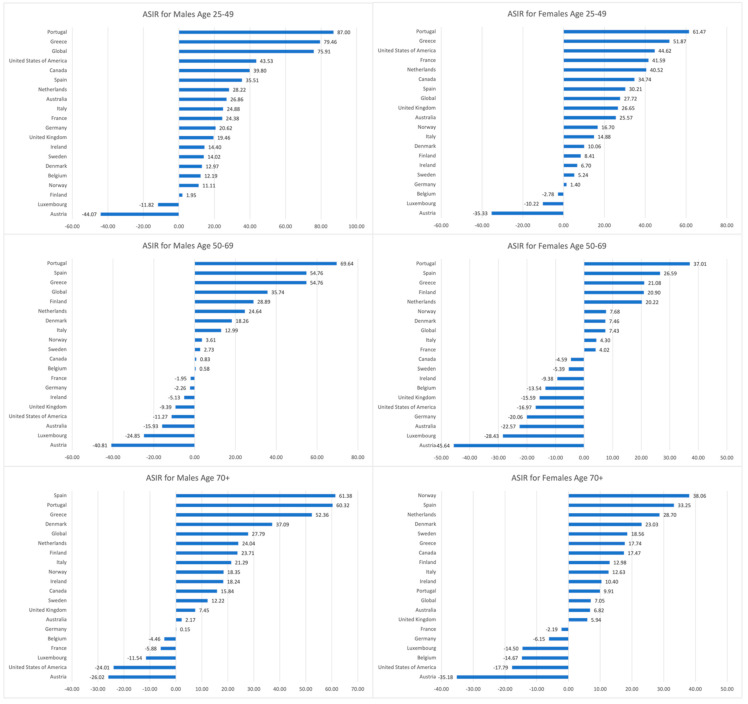
The percentage change in the age-stratified incidence rates (ASIR) of EU15+ countries and global rates between 1990 and 2019 for males and females in age groups 25–49, 50–69, and 70+ years.

ASIRs decreased in five and six countries for males and females greater than 70 years, respectively. There were five common countries between both genders of the greater than 70 age group that exhibited a decrease in ASIR, including Austria (−26.02% males, −35.18% females), Belgium (−4.46% males, −14.67% females), France (−5.88% males, −2.19% females), Luxembourg (−11.54% males, −14.40% females), and the United States (−24.01% males, −17.79% females). Germany experienced a reduced ASIR in females over 70 but an increase in their male counterparts (+0.15% males, −6.15% females). All other countries reported increased ASIRs for males and females over 70 years. Figure 2 outlines the percentage changes in ASIRs of EU15 countries and globally between 1990 and 2019.

### 3.2. Trends in CRC ASMRs, 1990–2019

**Figure 3:**
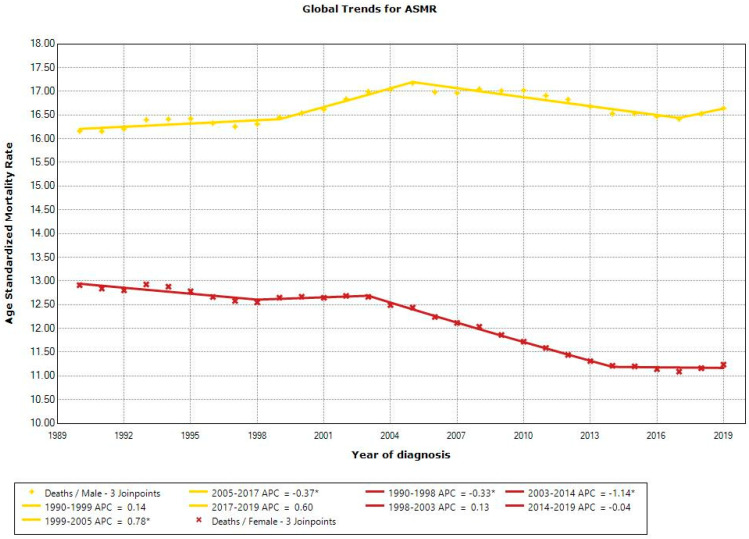
Joinpoint analysis of age-standardized mortality rates for males and females at global level between 1990 and 2019. * indicates a significant *p*-value of <0.05.

Throughout the study period, there was a decrease in global ASMR for all age groups, with females aged 50–69 experiencing the greatest reduction (−17.70%), while global ASMR increased for all age groups in males, with the 25–49 age group exhibiting the greatest increase (+22.23%) (Supplementary Table S1). The Joinpoint analysis of the ASMRs of males at the global level showed no significant change from 1990 to 2019, whereas the ASMRs of females at the global level showed a significant decrease (Figure 3).

The ASMRs for males in a majority of EU15+ countries across all age groups displayed a discordant trend relative to male ASMR at the global level. There were two common countries among all male age groups that reported increased ASMR: Greece and Portugal. Additionally, the U.S. exhibited increased incidence in males aged 25–49 (+16.84%), whereas Spain reported an ASMR increase in males greater than 70 years (+25.87%). All other countries across all male age groups reported ASMR decreases between 1990 and 2019.

**Figure 4:**
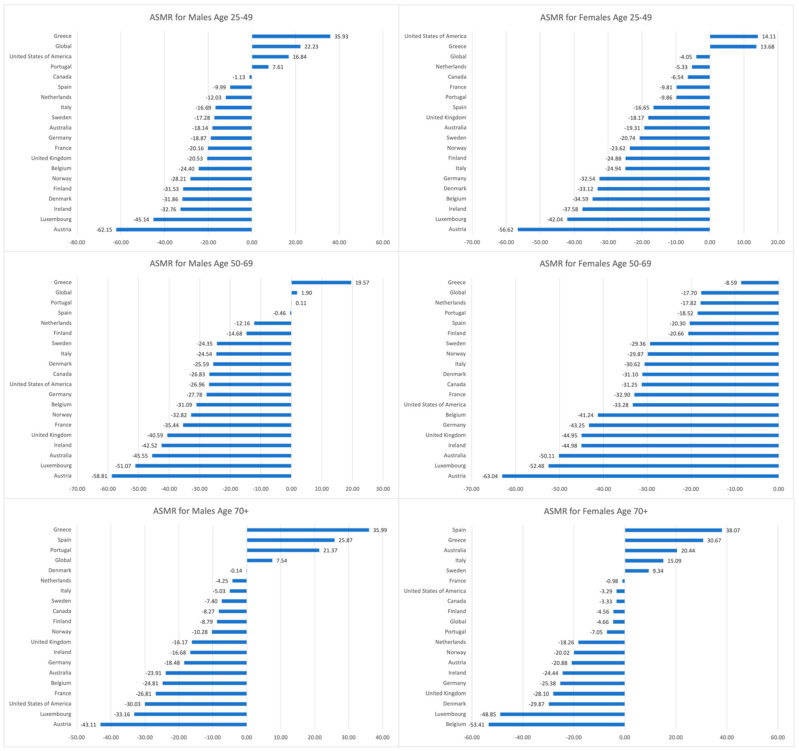
Percentage change in the age-stratified mortality rates (ASMR) of EU15+ countries and global rates between 1990 and 2019 for males and females in age groups 25–49, 50–69, and 70+ years.

The ASMRs for females revealed a similar trend to their male counterparts, with a majority of countries reporting decreases. All countries in the 25–49 female group reported decreased ASMRs except for the U.S. (+14.11%) and Greece (+13.68%). All countries reported decreased ASMRs in females aged 50–69. Similarly, all countries with females greater than 70 years reported decreased ASMRs except for Spain (+38.07%), Greece (+30.67%), Australia (+20.44%), Italy (+15.09%), and Sweden (+9.34%). Austria consistently reported the biggest decrease in ASMRs across both genders and all age groups except for females greater than 70 years. Figure 4 outlines the percentage change in the ASMRs of EU15+ countries and globally between 1990 and 2019.

### 3.3. Trends in CRC MIRs, 1990–2019

**Figure 5:**
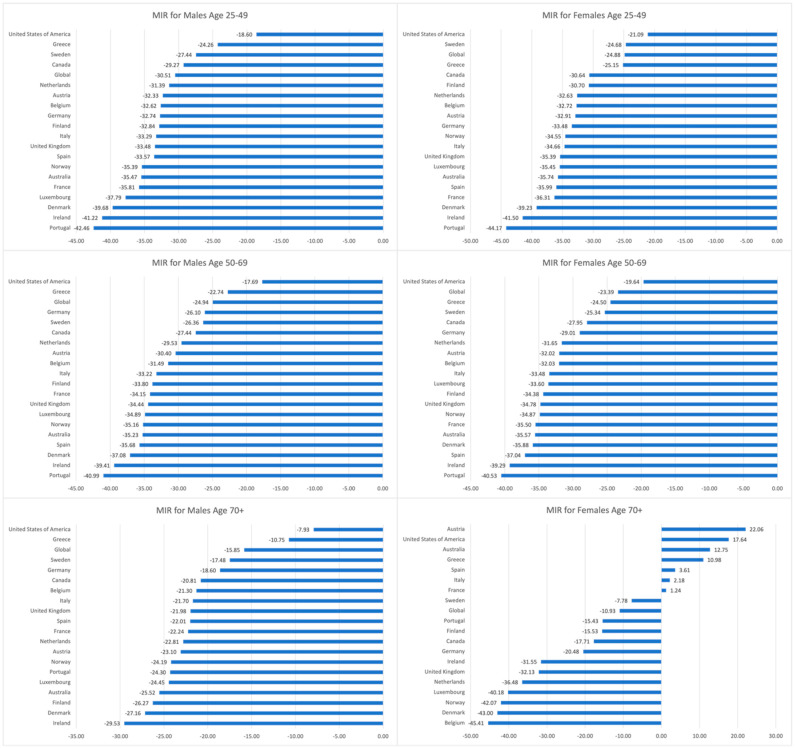
Percentage change in the mortality-to-incidence ratios (MIR) of EU15+ countries and global ratios between 1990 and 2019 for males and females in age groups 25–49, 50–69, and 70+ years.

Across the study period, there was a decrease in MIR globally for both males and females of all age groups, with the greatest global decrease observed in the 25–49 age group for both genders (−30.51% males, −24.88% females) (Supplementary Table S1). All EU15+ countries exhibited a decline in MIRs for males of all age groups. Females followed a similar trend; however, females greater than 70 years reported several countries with increasing MIRs, including Austria (+22.06%), the U.S. (+17.64%), Australia (+12.75%), Greece (+10.98%), Spain (+3.61%), Italy (+2.18%), and France (+1.24%). All EU15+ countries for females aged 25–49 and 50–69 reported decreased MIRs. Portugal exhibited the greatest MIR reduction among males and females aged 25–49 and 50–69. Interestingly, the U.S. exhibited the smallest MIR reduction in every group except for females aged 70+. Figure 5 outlines the change in the MIRs of EU15+ countries and globally between 1990 and 2019.

## 4. Discussion

This observational analysis explored CRC incidence and mortality rates from 1990 to 2019 within the EU15+ nations and on a global scale. A notable rise in CRC incidence was identified worldwide, particularly among individuals aged 25–49 of both genders, aligning with the American Cancer Society’s 2018 directive to initiate CRC screening from 45 rather than 50 in the U.S. [21]. Remarkably, mortality rates diminished in the face of this escalating incidence in most countries and age groups; however, a minority exhibited increasing mortality.

Our investigation, covering North American and EU15+ developed countries, revealed an upward trend in ASIRs in nations such as Portugal, Greece, Spain, Netherlands, Italy, Denmark, Norway, Finland, and globally. In contrast, Austria and Luxembourg saw reductions in ASIRs across all age groups. This aligns with Melina Arnold et al.’s study, which categorized nations by the Human Development Index (HDI) [7]. Countries with the highest HDI, including the U.S., Austria, France, and Australia, demonstrated reductions in both incidence and mortality rates. However, most EU countries with an above-average HDI showed increased incidence but decreased mortality rates [7]. Our findings support these results, showing that absolute incidence among the 50–69 and over-70 age groups still surpasses that of the 25–49 age group (Supplementary Table S1). Despite this, the 25–49 age group experienced the most significant incidence increase across EU15+ nations.

There is potential for the increasing incidence rates of CRC to be attributed to exposures inherent to a “Western” lifestyle [7]. Previous studies have shown a correlation between a variety of risk factors and the development of CRC. Multiple nonmodifiable and modifiable risk factors were identified. Nonmodifiable risk factors predominately consisted of age, gender, race/ethnicity, high-risk genetic syndromes, a personal history of polyps, IBD, and diabetes. Modifiable risk factors included smoking, alcohol use, obesity, the amount of physical activity, diet, medication use, and potential environmental exposures [22]. Furthermore, growing global awareness of colon cancer and the application of CRC screening methodologies may temporarily increase newly diagnosed CRC cases, but these practices could potentially reduce long-term incidence rates as more precancerous lesions are identified and removed [23]. For instance, the U.S., which initiated population-wide screening in the 1990s, exhibited a decrease in incidence for both age groups above 50. Similarly, Austria had records of opportunistic gFOBT screening dating back to 1980, and Luxembourg introduced gFOBT/colonoscopy screening in 2005 [24,25]. Both countries began offering colonoscopy as a primary screening modality between 2013 and 2015, with utilization rates peaking at 52–68.8% in Austria and 49% in Luxembourg. By contrast, Greece lagged with a rate of only 15% [4,26]. Moreover, countries such as Greece, Norway, and Portugal, which had no CRC screening programs as of 2013, have reported a significant surge in incidence rates [26]. Our database did not include detailed population screening data for each country. However, other studies have reported on recent trends in CRC screening usage across many EU countries [27]. One study assessed the utilization of fecal tests and colonoscopies between 2018 and 2020 in 29 EU countries, concluding that screening rates varied widely, ranging from less than 10% to over 70% of the population aged 50–74 [28]. The escalation of global awareness and the implementation of CRC screening programs may induce a drop in incidence rates in the coming decades, underscoring the necessity for a follow-up investigation of global trends.

Despite the rising incidence, both ASMRs and MIRs displayed a downward trend in most EU15+ nations, with several outliers appreciated. Along with advances in therapeutics and management, CRC screening likely contributed to this reduction in mortality, as early detection is crucial for improving CRC prognosis. Although many EU countries had screening practices in place, there was significant variance in the modalities used, and changes in these modalities were often seen over the study period. Notably, many EU CRC screening practices increased fecal testing following the COLONPREV trial’s initial findings in 2012 [29]. Regardless of the screening modality used, extensive CRC screening has demonstrated a contributory role in reducing mortality in both the EU and the U.S. [30,31]. Additionally, advancements in surgical techniques, adjuvant chemotherapy, radiotherapy, and nutritional support have also been instrumental in reducing mortality rates [32]. Trends in incidence and mortality were generally consistent between males and females. A slight increase in mortality was observed in the male demographic compared to their female counterparts. Previous studies have shown that hormonal status in younger women plays an important role in development, pathogenesis, and prognostication, leading to better survival in females [33].

Both genders within the 25–49 age group exhibited the highest increased incidence, suggesting the burden of disease onset is shifting towards younger individuals. This unique population of patients developing CRC under the age of 50, identified as early-onset CRC (EOCRC), often presents with CRC at a more advanced stage, with a predominance on the left side, and may exhibit different molecular characteristics compared to older-onset cases [34]. A prior U.S. study showed a similar trend, with more individuals being diagnosed at increasingly advanced cancer stages [1]. The cause behind this remains unknown; it is possible that modern Western lifestyle trends, such as reduced physical activity, increased obesity, diabetes mellitus (DM), cigarette smoking, and moderate-to-heavy alcohol consumption, could be driving the onset of CRC towards younger ages by altering cancer etiology [1,34]. Other potential factors related to newer and Western lifestyles—including processed meat, fast food, sugary drinks, a non-Mediterranean Western diet, insecticide exposure, genetically modified foods, radiation exposure, and antibiotic exposure in childhood or in utero—could also be contributing to the increasing CRC incidence and warrant further epidemiological research [34,35]. Overall, different clinico-pathological and molecular findings in EOCRC support the observation that it may be a distinct disease entity, posing unique challenges in management regarding fertility, pregnancy, sexual health, financial toxicity, and long-term survivorship [34,36].

Interestingly, despite the overall decrease in mortality across all age groups, females over the age of 70 exhibited the largest increase in mortality in some countries, including Spain, Greece, Australia, Italy, and Sweden. This trend may be attributed to elderly females being more likely to have aggressive tumor types, particularly on the right side, which can lead to poorer nutrition, a higher likelihood of overlooked flat-type tumors during colonoscopy, and a delayed diagnosis [37]. Additionally, this demographic is more prone to developing biologically aggressive tumors, such as those with the BRAF V600E mutation. Moreover, estrogen exposure has been found to be a protective factor against microsatellite instability, and the lack of estrogen in elderly females increases the risk of microsatellite instability–high colon cancer [24]. This may explain why the global increase in male incidence is almost twice as high as that of females, yet elderly females demonstrate a relatively increased incidence compared to males (Figure 2). Austria and the U.S. ranked high on the mortality-to-incidence ratio (MIR), likely due to a worse prognosis despite decreasing incidence. Future research should investigate screening and treatment strategies based on sex disparities to address this issue effectively.

## 5. Limitations

The primary strength of the GBD database lies in its estimation method, which integrates multiple sources, unlike single-source registries. Even comprehensive databases, such as SEER, only cover a small portion of the country (48%) [38]. Despite the strengths, there are multiple limitations inherent to the design of this study. One is potential reporting bias and potential inaccuracies regarding the database data [16,39]. These limitations related to GBD datasets have been previously noted by our studies and collaborators. GBD study data are based on robust estimation, with compilations of data from multiple sources as mentioned above. Higher-resource countries are likely to have more accurate and thus representative data of their populace than lower-income countries for both incidence and mortality reporting. For instance, multiple studies have suggested high rates of death certification inaccuracies in a variety of geographical regions [40,41]. Furthermore, CRC screening patterns have a large influence on CRC incidence and mortality trends, as a diagnosis typically requires a higher resource setting. Lower resource areas are more likely to have reduced, unrepresentative incidence rates due to this limitation. Another limitation is the presence of alterations in data coding systems and country-specific practices during the study period, markedly a shift from the use of ICD 9 to ICD 10. However, the GBD authors map mortalities to cause-of-death lists, adjusting by such to the different coding systems. There is also the existence of variability in the reliability of death certification within and across countries, with worldwide errors in death certification ranging from 39 to 61% [40,41,42]. To balance the under-registration, the GBD uses garbage-code distribution algorithms and corrections that relate to deaths resulting from poorly defined diagnoses or those that cannot be the single underlying cause of death [12,13]. Lastly, due to the lack of availability through this database, we could not evaluate colorectal cancer subtypes based on location, staging, or histo-pathology, which can be a future hypothesis and study.

## 6. Conclusions

Between 1990 and 2019, the incidence of CRC among men and women younger than 50 years old increased globally and in a majority of EU15+ countries. This increased incidence was not unique to EU15+ countries with “Western lifestyle” risk factors. Reassuringly, a majority of EU15+ countries exhibited a decreasing mortality rate over this 20-year period. However, the U.S. exhibited a relatively high increase in CRC incidence and mortality in the youngest age demographics, which is concerning for increased predisposition to CRC development. The incidence among both genders aged 50–69 is decreasing in most EU15+ countries, including the U.S. This is potentially related to the success of CRC screening in these countries. Lastly, the mortality and MIRs are decreasing in most demographics and geographic regions from 1990 to 2019, which likely reflects the improvement in cancer management since 1990.

## Data Availability

The data presented in this study are openly available in Global Burden of Disease at https://vizhub.healthdata.org/gbd-results/ (accessed on 4 May 2024), reference number [24].

## Supplementary Materials

The following supporting information can be downloaded at: https://www.mdpi.com/article/10.3390/cancers16122277/s1, Table S1: Male and female age-stratified incidence rates (ASIRs), age-stratified mortality rates (ASMRs), mortality-to-incidence ratios (MIR), and disability-adjusted life years (DALY), with associated percentage changes, for CRC globally and from EU15+ countries between 1990 and 2019.
