# Supplementary material for: Does a “Western Lifestyle” Confer a Higher Burden of Colorectal Cancer? A Comparison of EU15+ Countries versus Global Trends between 1990 and 2019
Source: Cancers (Basel). 2024 Jun 19;16(12):2277. doi: 10.3390/cancers16122277 (PMC11201493; doi:10.3390/cancers16122277)
Supplement: Supplementary file 1 [file cancers-16-02277-s001.zip › cancers-3024772-supplementary.pdf]

Supplementary Materials:

Supplementary Table S1. Male and female age-stratified incidence rates (ASIRs), age-stratified mortality rates (ASMRs), mortality-to-incidence ratios (MIR), and disability-adjusted life years (DALY), with associated percentage changes, for CRC globally and from EU15+ countries between 1990 and 2019.

TCountry/Region	ASIR			ASMR			MIR			DALY		
Males 25-49	1990	2019	% Change	1990	2019	% Change	1990	2019	% Change	1990	2019	% Change
Global	5.52	9.70	75.91	2.96	3.62	22.23	0.54	0.37	-30.51	144.38	176.04	21.92
Australia	12.30	15.61	26.86	4.22	3.46	-18.14	0.34	0.22	-35.47	201.81	170.83	-15.35
Austria	16.00	8.95	-44.07	5.94	2.25	-62.15	0.37	0.25	-32.33	282.10	107.90	-61.75
Belgium	8.64	9.69	12.19	3.52	2.66	-24.40	0.41	0.27	-32.62	168.32	127.44	-24.28
Canada	11.24	15.71	39.80	3.28	3.24	-1.13	0.29	0.21	-29.27	158.74	160.19	0.92
Denmark	9.75	11.02	12.97	4.43	3.02	-31.86	0.45	0.27	-39.68	210.33	145.15	-30.99
Finland	8.03	8.18	1.95	3.06	2.10	-31.53	0.38	0.26	-32.84	147.60	102.74	-30.39
France	9.24	11.49	24.38	3.88	3.09	-20.16	0.42	0.27	-35.81	185.47	148.81	-19.77
Germany	10.25	12.36	20.62	4.06	3.30	-18.87	0.40	0.27	-32.74	192.06	159.48	-16.96
Greece	5.46	9.80	79.46	2.08	2.83	35.93	0.38	0.29	-24.26	100.19	135.36	35.10
Ireland	9.88	11.30	14.40	4.17	2.80	-32.76	0.42	0.25	-41.22	197.06	135.81	-31.08
Italy	11.11	13.87	24.88	3.91	3.26	-16.69	0.35	0.23	-33.29	187.72	156.44	-16.66
Luxembourg	10.83	9.55	-11.82	4.56	2.50	-45.14	0.42	0.26	-37.79	217.12	122.29	-43.68
Netherlands	10.73	13.75	28.22	3.86	3.40	-12.03	0.36	0.25	-31.39	184.12	163.02	-11.46
Norway	10.17	11.30	11.11	4.09	2.94	-28.21	0.40	0.26	-35.39	196.48	142.71	-27.36
Portugal	10.58	19.79	87.00	4.66	5.02	7.61	0.44	0.25	-42.46	225.54	239.85	6.34
Spain	10.83	14.67	35.51	3.99	3.59	-9.99	0.37	0.24	-33.57	194.66	172.37	-11.45
Sweden	8.33	9.50	14.02	3.29	2.72	-17.28	0.39	0.29	-27.44	157.13	131.89	-16.06
United Kingdom	10.25	12.25	19.46	4.22	3.35	-20.53	0.41	0.27	-33.48	199.80	163.95	-17.94
United States of America	11.55	16.57	43.53	3.66	4.28	16.84	0.32	0.26	-18.60	179.20	207.90	16.02

Country/Region	ASIR			ASMR			MIR			DALY		
Females 25-49	1990	2019	% Change	1990	2019	% Change	1990	2019	% Change	1990	2019	% Change
Global	4.97	6.35	27.72	2.66	2.55	-4.05	0.54	0.40	-24.88	130.01	123.65	-4.89
Australia	11.49	14.43	25.57	3.80	3.07	-19.31	0.33	0.21	-35.74	183.13	152.09	-16.95
Austria	12.28	7.94	-35.33	4.09	1.77	-56.62	0.33	0.22	-32.91	196.46	85.74	-56.36
Belgium	9.16	8.90	-2.78	3.46	2.26	-34.59	0.38	0.25	-32.72	165.35	108.86	-34.16
Canada	9.88	13.31	34.74	2.95	2.76	-6.54	0.30	0.21	-30.64	143.25	135.86	-5.16
Denmark	9.54	10.50	10.06	4.09	2.73	-33.12	0.43	0.26	-39.23	191.44	131.23	-31.45
Finland	7.24	7.85	8.41	2.43	1.82	-24.88	0.34	0.23	-30.70	116.55	88.59	-23.99
France	7.26	10.27	41.59	2.83	2.55	-9.81	0.39	0.25	-36.31	136.57	122.94	-9.98
Germany	9.56	9.70	1.40	3.67	2.48	-32.54	0.38	0.26	-33.48	173.87	120.13	-30.91

Greece	6.29	9.55	51.87	2.21	2.51	13.68	0.35	0.26	-25.15	106.94	120.37	12.57
Ireland	9.96	10.63	6.70	3.86	2.41	-37.58	0.39	0.23	-41.50	183.29	117.64	-35.82
Italy	11.26	12.94	14.88	3.71	2.78	-24.94	0.33	0.22	-34.66	177.91	134.07	-24.64
Luxembourg	9.20	8.26	-10.22	3.54	2.05	-42.04	0.39	0.25	-35.45	171.59	100.41	-41.48
Netherlands	10.13	14.23	40.52	3.40	3.22	-5.33	0.34	0.23	-32.63	163.36	154.70	-5.30
Norway	10.33	12.06	16.70	3.74	2.86	-23.62	0.36	0.24	-34.55	179.93	138.02	-23.29
Portugal	10.08	16.27	61.47	4.16	3.75	-9.86	0.41	0.23	-44.17	202.98	181.58	-10.54
Spain	10.05	13.08	30.21	3.55	2.96	-16.65	0.35	0.23	-35.99	172.60	142.17	-17.63
Sweden	8.72	9.18	5.24	3.18	2.52	-20.74	0.36	0.27	-24.68	151.25	121.30	-19.80
United Kingdom	8.84	11.19	26.65	3.42	2.80	-18.17	0.39	0.25	-35.39	162.36	137.03	-15.60
United States of America	9.71	14.04	44.62	2.98	3.40	14.11	0.31	0.24	-21.09	145.43	166.48	14.48

**Country/Region**	**ASIR**			**ASMR**			**MIR**			**DALY**		
**Males 50-69**	**1990**	**2019**	**% Change**	**1990**	**2019**	**% Change**	**1990**	**2019**	**% Change**	**1990**	**2019**	**% Change**
Global	61.42	83.37	35.74	33.43	34.06	1.90	0.54	0.41	-24.94	988.15	1018.61	3.08
Australia	175.30	147.38	-15.93	71.93	39.17	-45.55	0.41	0.27	-35.23	2112.32	1181.50	-44.07
Austria	186.76	110.53	-40.81	84.93	34.99	-58.81	0.45	0.32	-30.40	2482.11	1041.41	-58.04
Belgium	125.44	126.17	0.58	59.65	41.11	-31.09	0.48	0.33	-31.49	1710.44	1211.54	-29.17
Canada	160.90	162.24	0.83	56.57	41.39	-26.83	0.35	0.26	-27.44	1669.93	1247.84	-25.28
Denmark	121.48	143.67	18.26	64.73	48.17	-25.59	0.53	0.34	-37.08	1851.53	1413.74	-23.64
Finland	79.12	101.97	28.89	36.78	31.38	-14.68	0.46	0.31	-33.80	1072.63	915.69	-14.63
France	136.42	133.76	-1.95	66.58	42.99	-35.44	0.49	0.32	-34.15	1907.04	1265.57	-33.64
Germany	132.72	129.71	-2.26	59.70	43.11	-27.78	0.45	0.33	-26.10	1781.02	1285.32	-27.83
Greece	66.99	103.68	54.76	29.75	35.57	19.57	0.44	0.34	-22.74	872.37	1048.32	20.17
Ireland	153.91	146.02	-5.13	75.28	43.27	-42.52	0.49	0.30	-39.41	2176.34	1292.95	-40.59
Italy	132.58	149.80	12.99	53.62	40.46	-24.54	0.40	0.27	-33.22	1565.84	1220.12	-22.08
Luxembourg	138.38	104.00	-24.85	67.38	32.97	-51.07	0.49	0.32	-34.89	1962.33	982.05	-49.96
Netherlands	137.25	171.07	24.64	57.77	50.75	-12.16	0.42	0.30	-29.53	1683.05	1500.80	-10.83
Norway	131.70	136.46	3.61	62.42	41.93	-32.82	0.47	0.31	-35.16	1765.92	1234.68	-30.08
Portugal	108.34	183.79	69.64	58.06	58.13	0.11	0.54	0.32	-40.99	1680.35	1728.24	2.85
Spain	114.68	177.47	54.76	50.34	50.11	-0.46	0.44	0.28	-35.68	1459.74	1512.81	3.64
Sweden	101.59	104.36	2.73	45.88	34.71	-24.35	0.45	0.33	-26.36	1314.76	1015.92	-22.73
United Kingdom	144.93	131.32	-9.39	69.89	41.52	-40.59	0.48	0.32	-34.44	1999.87	1235.06	-38.24
United States of America	164.65	146.09	-11.27	58.87	42.99	-26.96	0.36	0.29	-17.69	1726.33	1308.82	-24.19

**Country/Region**	**ASIR**			**ASMR**			**MIR**			**DALY**		
**Females 50-69**	**1990**	**2019**	**% Change**	**1990**	**2019**	**% Change**	**1990**	**2019**	**% Change**	**1990**	**2019**	**% Change**
Global	48.39	51.98	7.43	26.55	21.85	-17.70	0.55	0.42	-23.39	782.96	654.13	-16.45
Australia	125.25	96.98	-22.57	49.15	24.52	-50.11	0.39	0.25	-35.57	1451.80	749.49	-48.38
Austria	118.95	64.66	-45.64	50.49	18.66	-63.04	0.42	0.29	-32.02	1422.77	562.80	-60.44

Belgium	93.39	80.74	-13.54	42.39	24.91	-41.24	0.45	0.31	-32.03	1215.73	744.65	-38.75
Canada	113.05	107.87	-4.59	40.13	27.59	-31.25	0.35	0.26	-27.95	1182.34	838.39	-29.09
Denmark	102.46	110.10	7.46	52.25	36.00	-31.10	0.51	0.33	-35.88	1508.95	1062.33	-29.60
Finland	59.29	71.68	20.90	26.18	20.77	-20.66	0.44	0.29	-34.38	755.91	616.73	-18.41
France	77.73	80.85	4.02	36.51	24.50	-32.90	0.47	0.30	-35.50	1049.58	729.29	-30.52
Germany	98.51	78.75	-20.06	43.70	24.80	-43.25	0.44	0.31	-29.01	1266.48	742.65	-41.36
Greece	56.37	68.25	21.08	23.76	21.72	-8.59	0.42	0.32	-24.50	701.81	654.21	-6.78
Ireland	102.91	93.26	-9.38	47.35	26.05	-44.98	0.46	0.28	-39.29	1383.86	782.60	-43.45
Italy	89.42	93.27	4.30	35.40	24.56	-30.62	0.40	0.26	-33.48	1038.19	749.79	-27.78
Luxembourg	98.59	70.56	-28.43	45.60	21.67	-52.48	0.46	0.31	-33.60	1327.16	651.58	-50.90
Netherlands	106.09	127.54	20.22	42.98	35.32	-17.82	0.41	0.28	-31.65	1255.26	1060.04	-15.55
Norway	103.40	111.34	7.68	45.86	32.16	-29.87	0.44	0.29	-34.87	1308.15	960.69	-26.56
Portugal	67.41	92.36	37.01	35.41	28.85	-18.52	0.53	0.31	-40.53	1030.85	865.46	-16.04
Spain	77.72	98.38	26.59	32.42	25.84	-20.30	0.42	0.26	-37.04	953.26	798.76	-16.21
Sweden	88.93	84.14	-5.39	38.16	26.96	-29.36	0.43	0.32	-25.34	1098.39	793.77	-27.73
United Kingdom	104.48	88.19	-15.59	48.60	26.76	-44.95	0.47	0.30	-34.78	1390.98	806.46	-42.02
United States of America	113.02	93.84	-16.97	40.29	26.88	-33.28	0.36	0.29	-19.64	1180.36	818.75	-30.64

**Country/Region**	**ASIR**			**ASMR**			**MIR**			**DALY**		
**Males 70+**	**1990**	**2019**	**% Change**	**1990**	**2019**	**% Change**	**1990**	**2019**	**% Change**	**1990**	**2019**	**% Change**
Global	207.10	264.66	27.79	142.93	153.70	7.54	0.69	0.58	-15.85	2160.06	2230.84	3.28
Australia	450.76	460.55	2.17	243.52	185.31	-23.91	0.54	0.40	-25.52	3771.96	2655.40	-29.60
Austria	513.46	379.87	-26.02	343.04	195.16	-43.11	0.67	0.51	-23.10	4985.46	2717.13	-45.50
Belgium	463.33	442.65	-4.46	320.19	240.75	-24.81	0.69	0.54	-21.30	4526.87	3231.48	-28.62
Canada	427.22	494.88	15.84	213.81	196.13	-8.27	0.50	0.40	-20.81	3267.47	2818.52	-13.74
Denmark	371.82	509.71	37.09	269.01	268.63	-0.14	0.72	0.53	-27.16	4000.62	3886.37	-2.86
Finland	265.60	328.57	23.71	170.79	155.79	-8.79	0.64	0.47	-26.27	2549.98	2228.14	-12.62
France	459.61	432.60	-5.88	327.75	239.87	-26.81	0.71	0.55	-22.24	4568.80	3109.41	-31.94
Germany	456.97	457.64	0.15	301.29	245.60	-18.48	0.66	0.54	-18.60	4288.55	3350.82	-21.87
Greece	244.54	372.59	52.36	158.54	215.60	35.99	0.65	0.58	-10.75	2281.93	2804.55	22.90
Ireland	433.00	511.98	18.24	291.90	243.21	-16.68	0.67	0.48	-29.53	4443.61	3493.13	-21.39
Italy	384.22	466.01	21.29	235.51	223.67	-5.03	0.61	0.48	-21.70	3464.90	3070.31	-11.39
Luxembourg	479.75	424.40	-11.54	330.14	220.65	-33.16	0.69	0.52	-24.45	4844.22	3049.84	-37.04
Netherlands	451.91	560.53	24.04	278.25	266.42	-4.25	0.62	0.48	-22.81	4061.57	3805.40	-6.31
Norway	418.41	495.19	18.35	272.90	244.84	-10.28	0.65	0.49	-24.19	4010.39	3407.94	-15.02
Portugal	300.86	482.35	60.32	244.21	296.40	21.37	0.81	0.61	-24.30	3639.99	3993.80	9.72
Spain	364.25	587.84	61.38	232.66	292.85	25.87	0.64	0.50	-22.01	3402.69	3878.36	13.98
Sweden	339.72	381.22	12.22	213.32	197.53	-7.40	0.63	0.52	-17.48	3119.32	2762.03	-11.45
United Kingdom	429.27	461.24	7.45	276.83	232.07	-16.17	0.64	0.50	-21.98	4134.55	3193.11	-22.77

United States of America	473.76	360.03	-24.01	225.56	157.82	-30.03	0.48	0.44	-7.93	3419.59	2257.46	-33.98

Country/Region	ASIR			ASMR			MIR			DALY		
**Females 70+**	**1990**	**2019**	**% Change**	**1990**	**2019**	**% Change**	**1990**	**2019**	**% Change**	**1990**	**2019**	**% Change**
Global	169.42	181.36	7.05	121.82	116.14	-4.66	0.72	0.64	-10.93	1708.61	1523.19	-10.85
Australia	325.90	348.13	6.82	181.80	218.96	20.44	0.56	0.63	12.75	2567.75	1934.85	-24.65
Austria	338.90	219.67	-35.18	232.36	183.84	-20.88	0.69	0.84	22.06	3129.40	1540.05	-50.79
Belgium	353.68	301.81	-14.67	262.75	122.41	-53.41	0.74	0.41	-45.41	3307.86	2157.77	-34.77
Canada	301.89	354.64	17.47	163.14	157.70	-3.33	0.54	0.44	-17.71	2287.22	2010.70	-12.09
Denmark	296.11	364.31	23.03	227.38	159.46	-29.87	0.77	0.44	-43.00	3089.98	2825.86	-8.55
Finland	205.00	231.61	12.98	139.11	132.76	-4.56	0.68	0.57	-15.53	1908.63	1536.17	-19.51
France	286.60	280.33	-2.19	223.14	220.96	-0.98	0.78	0.79	1.24	2728.95	1969.05	-27.85
Germany	349.16	327.67	-6.15	238.73	178.14	-25.38	0.68	0.54	-20.48	3156.10	2307.74	-26.88
Greece	217.10	255.62	17.74	143.51	187.52	30.67	0.66	0.73	10.98	1980.23	1876.20	-5.25
Ireland	285.38	315.05	10.40	199.95	151.08	-24.44	0.70	0.48	-31.55	2794.66	2094.60	-25.05
Italy	260.93	293.88	12.63	167.89	193.22	15.09	0.64	0.66	2.18	2284.70	1871.47	-18.09
Luxembourg	340.77	291.36	-14.50	247.80	126.75	-48.85	0.73	0.44	-40.18	3287.80	2089.77	-36.44
Netherlands	321.08	413.22	28.70	211.58	172.95	-18.26	0.66	0.42	-36.48	2815.22	2737.97	-2.74
Norway	302.87	418.16	38.06	201.97	161.55	-20.02	0.67	0.39	-42.07	2732.11	2743.89	0.43
Portugal	212.40	233.44	9.91	189.35	176.00	-7.05	0.89	0.75	-15.43	2526.98	2053.75	-18.73
Spain	243.62	324.63	33.25	159.86	220.73	38.07	0.66	0.68	3.61	2175.70	2071.27	-4.80
Sweden	248.29	294.36	18.56	165.46	180.91	9.34	0.67	0.61	-7.78	2193.40	2129.61	-2.91
United Kingdom	310.92	329.39	5.93	214.73	154.32	-28.10	0.69	0.47	-32.13	2852.43	2253.94	-20.98
USA	343.36	282.26	-17.79	174.04	168.32	-3.29	0.51	0.60	17.64	2385.85	1690.17	-29.16
